# Somatoform disorders in out-patient psychiatric setting: An overview

**DOI:** 10.1192/j.eurpsy.2023.1445

**Published:** 2023-07-19

**Authors:** Y. A. Yaseen

**Affiliations:** Medicine, University of Duhok, Duhok, Iraq

## Abstract

**Introduction:**

The somatoform disorders are a group of psychiatric disorders that present with unexplained physical symptoms.

**Objectives:**

This study aimed at assessing the prevalence and risk factors of somatoform disorders (SD) and their types among patients attending a major psychiatric clinic in Duhok Governorate/Kurdistan Region of Iraq. Our secondary aim was to assess the common presenting symptoms of conversion disorder (CD).

**Methods:**

637 subjects were randomly selected from the outpatient psychiatric clinic at Azadi Teaching Hospital in Duhok Governorate/Kurdistan Region of Iraq. Structured Clinical Interview for DSM-IV Axis I Disorders-Patient Edition (Version 2.0) was applied to diagnose patients with SDs.

**Results:**

In our sample the prevalence of SD was 24%. CD comprised the vast majority of SD at 75.8%, followed by somatization disorder at 7.8% and undifferentiated SD at 5.2%. SD was most common (60.1%) in adolescents and young adults (ages 15-25 y.o.); (*p* <0.05), and female gender comprised most of the SD in our sample (75.8%; p <0.001)

Although, more than two-third of the cases were from lower educational levels (illiterate and primary educational level) (67.3%), more than fifty percent were married (52.3%), majority were housewives (39.2%) and more than half of the cases were from urban areas (52.3%), but no significant association were found between SD and educational level, marital status, occupation, and residence (p-values were 0.218, 0.659, 0.072, 0.090 respectively). Regarding the symptomatic presentation of CD, vast majority of the cases presented with pseudo-seizures which comprised (81%), followed by motor symptoms which comprised (17.2%), and sensory symptoms which constituted (1.7%) only.

**Conclusions:**

SD was highly prevalent among patients attending a major outpatient psychiatric clinic in Duhok Governorate/Kurdistan Region of Iraq, and CD was the most common presenting form of SD. Younger age (adolescents and young adults) and female gender comprised the majority of cases. Interestingly, the most common presenting symptom of CD in our sample was pseudo-seizures.

**Disclosure of Interest:**

None Declared

